# Escapism and Excessive Online Behaviors: A Three-Wave Longitudinal Study in Finland during the COVID-19 Pandemic

**DOI:** 10.3390/ijerph191912491

**Published:** 2022-09-30

**Authors:** Hannu Jouhki, Iina Savolainen, Anu Sirola, Atte Oksanen

**Affiliations:** 1 Faculty of Social Sciences, Tampere University, 33014 Tampere, Finland; 2 A-Clinic Foundation, Ratamestarinkatu 7 A, 00520 Helsinki, Finland; 3 Department of Social Sciences and Philosophy, University of Jyväskylä, 40014 Jyväskylä, Finland

**Keywords:** escapism, addiction, excessive behaviors, internet use, gambling, gaming

## Abstract

Excessive online behaviors refer to harmful or disproportionate use of digital network applications. Such behaviors are likely to be associated with escapist motives. Our aim was to analyze whether escapism predicts excessive gambling, excessive gaming, and excessive internet use over time. A longitudinal sample of Finnish residents aged 18–75 years (*n* = 1022, 51.27% male) was surveyed at three time points during the COVID-19 pandemic in 6-month intervals: April 2021 (Time 1), October–November 2021 (Time 2), and April–May 2022 (Time 3). Of the original Time 1 respondents, 66.80% took part in the surveys at both Time 2 and Time 3. All surveys included measures for excessive gambling (Problem Gambling Severity Index), excessive gaming (Internet Gaming Disorder Test), and excessive internet use (Compulsive Internet Use Scale). Three escapism-specific questions were used to construct a dedicated escapism variable. Socio-demographic variables, alcohol consumption, and psychological distress were used as controls. The study was conducted with multilevel regression analyses using hybrid models. Our research showed that escapism had strong within-person effects on excessive gambling, B = 0.18, *p* = 0.003; excessive gaming, B = 0.50, *p* < 0.001; and excessive internet use, B = 0.77, *p* < 0.001 over time. The between-person effect of escapism was demonstrated on excessive gaming B = 0.91, *p* < 0.001, and excessive internet use B = 0.61, *p* = 0.036. Adverse societal events and uncertain times can manifest in excessive online behaviors motivated by escapism, highlighting a need to focus prevention efforts on healthy coping methods.

## 1. Introduction

Excessive online behaviors refer to harmful or disproportionate amounts of time or money spent using digital network applications [1,2]. Some forms of excessive online behaviors such as disordered gambling and digital gaming are recognized as behavioral addictions in contemporary disease classifications [3,4]. In the broadest meaning, excessive online behaviors encompass all internet use. An evolving trend is the blurring of boundaries between different forms of online activities, such as the convergence of online gambling and digital gaming [5].

Escapism is the tendency to distract oneself from real-life problems. It can also be conceived as shutting meanings out of one’s mind and freeing oneself from self-awareness for a while [6]. Escapism has been identified as one of the key drivers behind online behaviors [7,8] in both adaptive and maladaptive ways [9]. Past research has indicated that escapism predicts excessive online behaviors irrespective of a competitive or collaborative context [10]. The desire to understand the drivers of excessive online behaviors has directed research toward the roles of motives [11] and social contexts [12].

The proliferation of social media has not only provided opportunities for networking and positive interaction but also laid ground for false or distorted self-presentation [13]. Research on adult Facebook users shows that people pursuing self-image goals on social media are more inclined toward social comparison and envy [14]. The way our communications environment has evolved during the past decades suggests that pressures for higher self-awareness and escapism have increased. In addition to intolerable self-awareness, escapism may stem from external pressures such as work, relationships, financial worries, other personal problems, or adverse events causing stress and anxiety.

Our longitudinal study analyzed whether escapism predicts excessive gambling, excessive gaming, and excessive internet use. Longitudinal evidence investigating escapism and excessive online behavior over time is scarce and our study aims to fill this gap in the current literature. Our study is grounded on escape theory; related motivation and reinforcement research; avoidance coping literature; and empirical studies of escapism, addiction, and excessive online behaviors. Escape theory suggests that people are likely to feel guilty and to blame themselves for falling short of their standards and personal values. The real versus ideal self-discrepancy may become so burdensome that the individual needs to seek relief from excessive self-awareness [15]. To escape from self-actualization effectively means forgetting the responsibilities, demands, obligations, criticisms, and judgments stemming from daily life and instead engaging in an activity where it is possible to suppress conscious thinking for a moment. At the time of its introduction, escape theory hypothesized that excessive self-awareness may lead to increased drinking, disordered eating, religious exercise, masochism, or even suicide [6]. Considering the massive progress in online devices, services, and applications over the past thirty years, it has become highly relevant to build our understanding on escapist behaviors in the present-day context and better understand how excessive online behaviors may serve escapist purposes.

The study of escapism is essential because it recognizes the importance of avoidance motives as opposed to positively reinforcing motives in explaining the development of harmful repetitive behaviors. Instead of being a pleasure-seeking drive that pushes individuals toward excessive behaviors, escapism represents relief-seeking that pushes people away from their daily problems and worries. Examining escapism in connection with excessive online behaviors introduces an alternative approach to the pursuit of gratification while building on prior research on escapism and addiction.

### 1.1. Escapism and Excessive Gambling

Escape-oriented gamblers tend to be motivated by a sense of relief, whereas players focused on task performance and action are more likely to be driven by reward winning [16]. Research has also identified a third category of gamblers: those with low emotional regulation needs [17,18].

Consistent evidence has shown that escapism is a risk factor for gambling problems [19,20,21,22,23,24]. However, when coupled with a relaxation component in both online and offline contexts, escapism tends to point toward non-problematic gambling as opposed to financially motivated players [25,26].

Earlier studies have suggested escapism motivation affects the choice of gambling type. In real-life contexts, escapist players tend to favor slot machines [27], but cards, dice games, and legal casinos also have been associated with escapism motivation [28].

### 1.2. Escapism and Excessive Gaming

Escapism has been recognized as an immersive motive of online gaming [29] and a strong predictor of problematic digital gaming [30]. Higher levels of escapism have been associated with longer times spent playing digital games [31], gaming disorder [32], and a preference for virtual stimuli over real-life ones [33]. The evidence of escapism’s connection to digital gaming is independent of player profile [34]. Escapism not only is a driver for adopting an alternative virtual reality for hedonic purposes but also explains continued online behaviors in competitive contexts such as eSports [10].

Although escapist gaming does not necessarily predict negative outcomes without underlying psychosocial problems [35], the social context of digital gaming does have a connection to mental health measures [12], and social alienation predicts problematic digital gaming [30]. Particularly for young people, digital gaming may serve as a means of escape from the self, to avoid controversies related to real-life emotional development [36].

Digital, or online, gaming has been a popular pastime during the COVID-19 pandemic, but a concurrent risk exists where gaming activities develop into unhealthy behavioral patterns [37]. A cross-sectional natural experiment examining the effects of self-isolation and quarantine on adults during the pandemic found that isolated online gamers showed stronger problematic gaming symptomology and loot box spending compared to those who were not isolated [38]. A fear of contamination by germs was also found to associate significantly with a higher rate of excessive gaming. A study interviewing young gamers found escape motives to be among the most popular reasons for engaging in online gaming during the pandemic [39]. A recent systematic review of the role of escape motives in problematic online gaming confirmed the role of escapism in predicting internet gaming disorder and concluded a need for longitudinal evidence exists [40].

### 1.3. Escapism and Excessive Internet Use

Previous research has well-established the connection between escapism and excessive internet use [41,42]. Evidence has also suggested that a continuum exists from psychological distress through escapism and internet addiction to harmful real-life consequences [43]. Furthermore, escapism has been found to mediate the association between deficient emotional regulation and problematic internet use [44] and between internet gaming involvement and problematic internet use [45].

Exploring the association between escapism and excessive internet use in a longitudinal setting including a period of large-scale social isolation should provide valuable insight on the role of social relations in excessive online behaviors. Research conducted among adolescents during the COVID-19 pandemic reported increased levels of online activity, which—combined with COVID-related worries—predicted higher levels of escapism [46]. 

In our analyses, we used the concepts of excessive gambling, excessive gaming, and excessive internet use when referring to harmful levels of online behaviors. Each concept included various other definitions such as problematic, disordered, compulsive, addictive, or appetitive behaviors, respectively.

### 1.4. This Study

This longitudinal study took place during social isolation caused by pandemic restrictions, when negative views of self and incentives to escape from self-awareness were arguably more commonplace. Taking note of the basic tenets of the escape theory, we formulated four hypotheses to investigate whether escapism predicts excessive online behaviors over time. We examined both within-person and between-person changes. Our modelling strategy allowed us to separate these effects, which is important because people with escapist tendencies typically report higher levels of excessive behaviors in comparison to non-escapists. Our hypotheses were:

**Hypothesis** **1** **(H1).** *Within-person changes in escapism predict excessive gambling over time.*

**Hypothesis** **2** **(H2).** *Within-person changes in escapism predict excessive gaming over time.*

**Hypothesis** **3** **(H3).** *Within-person changes in escapism predict excessive internet use over time.*

**Hypothesis** **4a–c** **(H4a–c).** *A higher between-person level of escapism is associated with (a) excessive gambling, (b) excessive gaming, and (c) excessive internet use.*

## 2. Methods

### 2.1. Participants and Procedure

The study participants were Finnish residents aged 18–75 years (*n* = 1530) who responded to a longitudinal survey conducted in three parts. Data were collected for the first time point (T1) in April 2021; the first follow-up survey (at Time 2, T2) took place in October–November 2021, with a response rate of 78% (*n* = 1200), and the second follow-up survey (at Time 3, T3) took place in April–May 2022, with a 72% response rate versus T2 (*n* = 1100). In total, 1022 respondents took part in all three waves, representing 66.80% of the original T1 respondents.

Data collection was accomplished in collaboration with a data provider company Norstat. The survey was designed by the authors and the researcher group, and Norstat customized it to their own platform. Participants were recruited from an online volunteer participant panel that Norstat administers. Norstat was responsible for data collection by sending an invite link to those volunteer participants in the online panel who matched the desired demographics of our study (i.e., 18- to 75-year-old Finnish residents in mainland Finland). Invitations to participate in the study were sent via email that included a link to the survey. Once data collection was completed, Norstat provided the anonymized data to the research group. Average response time was approximately 15 min (T1: 18.2 min, T2: 13.9 min, T3: 14.5 min). Participants were not directly offered incentives to partake in this study and their participation was completely voluntary, but Norstat does offer their panelists points and price draws that might help make taking surveys more attractive.

Participants taking part in all three waves (*n* = 1022) were included in this study. They were 51.27% male, 48.43% female, and 0.03% other gender. They were from all areas of mainland Finland: 36.30% were from the Helsinki–Uusimaa region, 20.25% were from Southern Finland, 24.27% were from Western Finland, and 19.18% were from Northern and Eastern Finland. A nonresponse analysis of the final sample participants (*n* = 1022) and T1 respondents (*n* = 1530) showed that the final sample participants were slightly older (49.50 years vs. 46.67 years, respectively). No major drop out based on gender, geographical area, income, education, marital status, or occupational status occurred. In comparison to the general population census figures from Statistics Finland, our sample did not have major deviations. Analysis of our variables of interest in T1 showed that mean figures for excessive behaviors and escapism were lower among our final sample (*n* = 1022) than original respondents (*n* = 1530): excessive gambling (1.15 vs. 1.31), excessive gaming (1.12 vs. 1.34), excessive internet use (7.95 vs. 8.79), and escapism (0.83 vs. 0.94). This could be explained by our final sample being slightly older than the original sample. As our analysis focuses only on the respondents who have taken part in all three waves the drop-out does not have impact on results.

The research group checked the data after each data collection phase in accordance with the data quality protocol stored on the Open Science Framework website prior to the data collection. Different integrity and data quality checks were conducted, for example, attention checks and patterned response checks. The Ethics Committee of the Tampere Region in Finland approved the study in March 2021. Participation was voluntary for the participants, and they were informed about the study’s aims.

### 2.2. Measures

#### 2.2.1. Dependent Variables

Excessive Gambling. Excessive gambling was measured using the nine-item Problem Gambling Severity Index (PGSI), a standardized measure for the self-assessment of problem gambling behaviors in the general population in non-clinical context [47,48]. The PGSI has been validated and widely used in Finnish population studies assessing problematic gambling [49,50,51,52]. The items included questions such as “[In the past 6 months], have you bet more than you could really afford to lose?”, “Have you borrowed money or sold anything to get money to gamble?”, and “Has your gambling caused any financial problems for you or your household?” Due to the 6-month intervals between our data collection, we slightly modified the measure to inquire about gambling-related experiences in the past 6 months. Each response was scored on a scale from 0 to 3 (0 = never, 1 = sometimes, 2 = most of the time, 3 = almost always), producing a range from 0 to 27, with higher scores pointing toward risky or problematic gambling. Internal consistency of the PGSI variable was excellent throughout the survey based on McDonald’s omega (T1: ω = 0.94, T2: ω = 0.93, T3: ω = 0.94; Table 1).

Excessive gaming. Excessive gaming was measured using the 10-item Internet Gaming Disorder Test (IGDT), an established gaming disorder screening scale with cross-culturally validated psychometric properties [53]. The IGDT has been used in prior studies in Finland and the Finnish version of the measure has been found to be psychometrically appropriate and suitable for assessing problematic gaming among adults [54,55,56]. Similar to what we did with the PGSI, we asked about gaming experiences in the past 6 months. Typical questions included “Have you ever [in the past 6 months] unsuccessfully tried to reduce the time spent on gaming?”, “Have you played a lot despite negative consequences?”, and “Have you risked or lost a significant relationship because of gaming?” The answers were recorded on a scale from 0 to 2 (0 = never, 1 = sometimes, 2 = often) constituting a minimum–maximum scale of 0–20, even though the range achieved in the study was 0–16. Higher scores indicated risky or problematic gaming. The IGDT variable had good internal consistency (T1: ω = 0.88, T2: ω = 0.90, T3: ω = 0.88; Table 1).

Excessive Internet Use. Excessive internet use was measured using the Compulsive Internet Use Scale (CIUS; [57]). In addition to its established position, concise structure, and ease of use, the CIUS scale has been psychometrically and culturally validated across different languages and populations [58,59]. Examples of the questions in the 14-item scale included “How often do you find it difficult to stop using the internet when you are online?”, “How often do you prefer to use the internet instead of spending time with others (e.g., friends, partner, children)?”, and “How often have you unsuccessfully tried to spend less time on the internet?” The response scale ranged from 0 to 4 (0 = never, 1 = seldom, 2 = sometimes, 3 = often, 4 = very often) and the total CIUS scores formed a minimum–maximum scale of 0–56, with higher scores indicating compulsive internet use. The range achieved in the study was 0–53. Internal consistency of the CIUS variable was excellent (T1: ω = 0.95, T2: ω = 0.95, T3: ω = 0.95; Table 1).

#### 2.2.2. Independent Variable

Escapism. To measure escapism in online context, we used the Motivation to Play in Online Games-Revised (MTPI-R), a version of the escapism subscale Hagström and Kaldo [60] developed based on the Motivation to Play in Online Games model [29]. The MTPI-R scale excludes positive aspects of escapism as theoretically and empirically unstable and focuses only on negative escapism. This means learning to repeat a behavior is not reinforced by its positive effects but instead by avoiding negative emotions or other burdensome aspects of real life. This was consistent with our approach because, in this study, escapism was considered the tendency to distract oneself from reality or real-life problems, which is why we treated escapism as a negative concept.

In addition to measuring the relationship between escapism and online gaming, the MTPI-R scale has shown a strong correlation between negative escapism and internet addiction, suggesting that it is a suitable measure of escapism with respect to multiple forms of online behaviors. We defined our escapism variable based on the corresponding three questions from the MTPI-R scale: “How often do you play so you can avoid thinking about some of your real-life problems or worries?”, “How often do you play in order to avoid real-life social encounters or situations?”, and “How often do you continue to play so that you won’t have to deal with everyday problems and issues?” The answers were recorded on a scale from 0 to 4 (0 = never, 1 = seldom, 2 = sometimes, 3 = often, 4 = always), producing a minimum–maximum scale of 0–12, with higher scores corresponding to higher escape motivation. Due to a low number of answers in the “4 = always” category, the range achieved in the study was 0–10.

The three escapism items appeared only once in the questionnaire after gambling- and gaming-related questions. Respondents were instructed to consider their gambling and gaming motives. Despite this, we considered our escapism measure as valid also for the investigations of excessive internet use. During the COVID-19 pandemic, the public gambling and gaming venues were closed and much of gambling and gaming took place online.

Internal consistency of the escapism variable was good across all time points (T1: ω = 0.84, T2: ω = 0.87, T3: ω = 0.88; Table 1). As the scale has not been previously validated in Finland, we ran additional confirmatory factor analysis (CFA) with maximum likelihood estimation on the scale. Factor loadings ranged, on average, from 0.71 to 0.89 in T1, from 0.77 to 0.92 in T2, and from 0.76 to 0.93 in T3. The fit of the estimated CFA met all the criteria of root mean squared error of approximation (RMSEA), standardized root mean squared residual (SRMR), comparative fit index (CFI), and Tucker–Lewis index (TLI) based on the criteria suggested by Hu and Bentler [61].

#### 2.2.3. Control Variables

Socio-demographic control variables were reported as binary categories (0/1) and included age greater than or equal to 40 years (69.37%), male gender (51.27%), educational level of a master’s degree or higher (21.72%), status of currently working (51.57%), income over EUR 4000 a month (17.03%), being in a relationship (59.78%), and having children (62.23%). Excessive drinking as measured by the Alcohol Use Disorders Identification Test-Concise (AUDIT-C) [62]. The AUDIT-C is an extensively used brief measure for risky drinking. It has been validated in several contexts and populations and has been found to be a reliable and optimal screen of risky alcohol use (e.g., [63,64,65]). The measure has been also extensively used in Finland over the years [66,67,68,69]. Lastly, psychological distress was measured by using the Mental Health Inventory scale (MHI-5) [70]. The MHI-5 is a short form of the original Medical Outcomes Study: Short Form Health Survey, measuring psychological and emotional wellbeing. The MHI-5 includes five items that screen for general mental health and mood disorders such as depression and anxiety. It is a commonly used and sensitive measure and has shown to perform well in different populations [71,72]. Both the AUDIT-C and MHI-5 variables served as continuous covariates.

### 2.3. Statistical Methods

The focus of our study was to analyze the longitudinal effects of reported escapism on excessive online behaviors during social isolation. The analyses were conducted with linear multilevel regression using hybrid models. A clear upside of hybrid models is their applicability to within-person effects over numerous time points while still allowing the examination of between-person differences [73]. To control within-person estimation bias, hybrid models were compared with standard fixed effect models. Stata 17.0 software (StataCorp LLC, College Station, TX, USA) was used throughout the analyses.

Descriptive statistics in Table 1 include a list of the study variables with the means and standard deviations across the three measured time points. Zero-order correlations of the variables at T1 and McDonald’s omega coefficients for all time points are specified for each variable. Socio-demographic control variables and their percentages are reported in the text. Table 2 reports the final multilevel hybrid regression models showing both within-person effects and between-person effects and the controls that were also interpreted as between-person effects. Our main variable of interest was escapism and especially its within-person effects on the dependent variables of excessive gambling, excessive gaming, and excessive internet use.

## 3. Results

Table 1 shows the changes in excessive online behaviors and escapism over time. The changes were not statistically significant, except for the increase in excessive gaming at T2 in comparison to T1 and the decrease in excessive gaming at T3 in comparison to T2. In addition, the control variables did not significantly change over time in comparison to the general levels. The zero-order correlations at T1 showed escapism was associated with excessive gambling, excessive gaming, excessive internet use, excessive drinking, and psychological distress (see Table 1).

Analyses based on the hybrid models indicated robust and consistent connections existed between escapism and excessive gambling, excessive gaming, and excessive internet use (Table 2). Escapism had strong and independent within-person effects on excessive gambling, B = 0.18, *p* = 0.003; excessive gaming, B = 0.50, *p* < 0.001; and excessive internet use, B = 0.77, *p* < 0.001. Hence, over time, increasing escapism experienced by the respondents led to higher levels of all studied excessive online behaviors.

The between-person effects demonstrated respondents with escapist tendencies differed from non-escapists across the three measured time points. People seeking escapist distractions from online content showed higher degrees of excessive gaming, B = 0.91, *p* < 0.001, and excessive internet use, B = 0.61, *p* = 0.036. Additionally, we found a between-person effect of escapism on excessive gambling, but this association was no longer statistically significant in the final model reported in Table 2.

Our observation of all covariates showed excessive gambling had within-person effects on excessive gaming, B = 0.21, *p* < 0.001, and excessive internet use, B = 0.53, *p* < 0.001. Likewise, the respondents who were more engaged in excessive gaming showed higher within-person effects on excessive gambling, B = 0.23, *p* < 0.001, and excessive internet use, B = 0.53, *p* < 0.001. Excessive internet use indicated higher within-person effects on excessive gambling, B = 0.04, *p* < 0.001, and excessive gaming, B = 0.04, *p* < 0.001. It is noteworthy that all different forms of excessive online behaviors in this study had independent statistically significant within-person effects on each other. Excessive drinking, B = 0.47, *p* < 0.001, and psychological distress, B = 0.13, *p* = 0.003, each had a within-person effect on excessive internet use, suggesting both increased alcohol consumption and higher distress independently led to greater excessive internet use. Drinking also predicted excessive gambling, B = 0.09, *p* = 0.038.

Regarding the between-person effects of the covariates, excessive gamblers reported more excessive gaming, B = 0.14, *p* < 0.001, whereas those more involved in excessive gaming showed higher propensities to excessive gambling, B = 0.59, *p* < 0.001, and excessive internet use, B = 1.30, *p* < 0.001. Excessive internet users reported more excessive gaming than non-excessive internet users, B = 0.04, *p* < 0.001. Furthermore, the respondents consuming more alcohol were more inclined to gamble excessively, B = 0.13, *p* = 0.001, whereas those experiencing psychological distress were less involved in excessive gaming, B = –0.04, *p* < 0.001, and more inclined toward excessive internet use, B = 0.47, *p* < 0.001.

Of the control variables, men reported more excessive gaming, B = 0.28, *p* < 0.001, and less excessive internet use, B = –1.90, *p* < 0.001. Older respondents showed a higher effect on excessive gambling, B = 0.02, *p* = 0.013, and a lower effect on excessive internet use, B = –0.14, *p* < 0.001. Higher education was linked with less excessive gambling, B = –0.30, *p* = 0.035, whereas having children indicated more excessive internet use, B = 0.97, *p* = 0.031.

## 4. Discussion

### 4.1. Main Findings

Building on escape theory and related motivation and escapism literature, this longitudinal study analyzed whether escapism predicts excessive gambling, excessive gaming, and excessive internet use over time. Our results demonstrate a sustained and robust connection between escapist motivation and excessive online behaviors. Escapism had strong and independent within-person effects on excessive gambling (H1), excessive gaming (H2), and excessive internet use (H3), indicating all studied online behaviors tend to increase when a person is undergoing a period of heightened escapism. Between-person effects of escapism were demonstrated on excessive gaming (H4b) and excessive internet use (H4c).

Contrary to the view that motivations are stable characteristics of a person, recognizing the changing nature of human drives over time is essential. Although satisfying a need normally takes away the appetite for that activity or substance for a short period, in the longer run, the same drive is likely to come back in a strengthened form [74]. This is consistent with our within-person findings, where the increasing levels of escapism experienced by the respondents led to higher levels of all studied excessive online behaviors.

Among other findings, this study demonstrated reciprocal within-person and between-person effects between excessive online gambling and excessive online gaming. This is concordant with past evidence on the neurobiological similarities between problematic gambling and internet gaming disorder [75]. The effects observed in our study could also be reflective of past research, indicating a larger convergence between online gambling and digital gaming (e.g., [76]). Digital gaming is becoming increasingly characterized by rewards and monetary prizes, whereas online gambling has incorporated social aspects and elements of skill. These structural similarities and converging features seem to appeal especially to problem gamblers [77]. Overall, our results contribute to the etiological debate around these excessive behaviors by presenting escapism as a common denominator behind both.

### 4.2. Theoretical and Empirical Implications

The present study contributes to the debate about the escape theory. Across three decades, the means of escape have evolved, largely by technological development. Before the age of the internet, the primary means of escaping intolerable self-awareness were intoxication, spirituality, or extreme forms of real-life behaviors. Today, online applications provide easily accessible content with which a connection to escapism has been shown by research and proven in the longitudinal setting of our paper. A theoretically solid reasoning would say that between-person effects of escapism in this study were mainly driven by individual levels of self-awareness, along the lines of the escape theory. Research on false Facebook-selves gives some support for the idea that escapist tendencies based on high self-awareness and an inflated self-image could be relatively stable characteristics [13].

The results of this three-wave study also emphasize the role of changing external circumstances as a driver for escapism. It is possible that excessive gamblers, gamers, and internet users might have been managing and coping with worries about the COVID-19 pandemic by escaping the real-life challenges to excessive online behaviors [38]. It is logical to speculate that the adaptation to changing pandemic circumstances was most likely reflected by the within-person effects of escapism in our study. Examining escapism in connection with excessive behaviors is relevant because it introduces an alternative approach to pleasure-seeking activities. A commonsense explanation for consuming a substance or engaging in a particular activity is the good feeling it produces. In contrast, doing something with an escapist motive involves avoiding negative emotions as opposed to pursuing good sensations. Given past research showing that avoiding something bad is a stronger psychological motive than gaining something good [78], a clear case exists to study escapism as a driver for excessive behaviors. Considering the appetitive nature of online applications, and how people use them for self-enhancement, an interesting topic for future research would be to what extent escapist use of social media platforms generates further escapism.

### 4.3. Practical Implications

Our findings lay out a new perspective to escapism, not only as a binary category but also as an alternating state of mind. Escapist coping is largely considered to provide a momentary relief instead of sustainable wellbeing, and it can lead to additional problems the excessive behaviors cause. Prevention and intervention efforts should focus on providing individuals with information, resources, and healthy ways to cope with uncertainty and adversity. One way to help people avoid excessive engagement in online behaviors is to strengthen their ability to stay present and to help them recognize when online behaviors turn into affect regulation.

### 4.4. Strengths and Limitations

The longitudinal design of the study is a key strength of this paper. The three time points of data collection conducted in 6-month intervals allowed us to monitor and analyze changes in excessive online behavior patterns combined with reported levels of escapism. The timing of the study further supports the contribution of our research, as it was carried out during a period of social isolation and intermittent lifts of COVID-19 restrictions. Therefore, the study provides insight into coping with uncertainty and stressful events. Another clear strength of the study is the use of hybrid regression models, which made it possible to simultaneously examine within-person and between-person effects of escapism on excessive online behaviors. The principal limitation of this study is related to location because the study was conducted entirely in Finland. Cultural differences in adapting to adverse societal circumstances, availability of online services, and public social and healthcare policies on isolation procedures may vary between countries. Additionally, it could be considered as a limitation that the context of our escapism measure was in gambling and gaming, and not in excessive internet use. However, as much of gambling and gaming took place online during the COVID-19 pandemic and even in normal circumstances, they increasingly move to online contexts, the escapism measure can be considered relevant for internet use as well. This is further highlighted in our results, showing an association between escapism and excessive internet use while controlling for the effects of excessive gambling and excessive gaming.

## 5. Conclusions

Our longitudinal study found that escapism predicted excessive online behaviors. The study was based on the escape theory and escapism literature, and it provided much needed longitudinal research evidence on the role of escapism in connection with online behaviors. The findings of the study indicate escapism is a highly relevant coping mechanism that could help us better understand risky online behaviors. In our study, the COVID-19 pandemic may have induced escape-motivated online behaviors, but it is equally possible that part of the perceived escapism originated from relatively stable psychological factors. It is meaningful to conclude that escapism is a multi-faceted phenomenon, that has both social and psychological origins.

## Figures and Tables

**Table 1:** Descriptive statistics of the main study variables.

					Zero Order Correlations at T1				
Continuous Variables	Range	T1, M (SD)	T2, M (SD)	T3, M (SD)	1	2	3	4	5
1. Escapism	0–10	0.83 (1.72)	0.82 (1.74)	0.80 (1.75)	1				
2. Excessive gambling	0–27	1.15 (3.02)	1.12 (2.98)	1.11 (3.03)	0.45 ***	1			
3. Excessive gaming	0–16	1.12 (2.36)	1.24 (2.52)	1.10 (2.29)	0.75 ***	0.53 ***	1		
4. Excessive internet use	0–53	7.95 (9.06)	8.20 (9.65)	7.85 (9.31)	0.51 ***	0.32 ***	0.49 ***	1	
5. Excessive drinking	0–12	3.54 (2.71)	3.46 (2.72)	3.42 (2.72)	0.09 **	0.17 ***	0.08 *	0.03	1
6. Distress	5–30	12.24 (4.67)	12.20 (4.58)	12.28 (4.428)	0.42 ***	0.24 ***	0.31 ***	0.43 ***	0.07 *

Note. *** *p* < 0.001, ** *p* < 0.01. * *p* < 0.05.

**Table 2:** Hybrid models showing within-person and between-person effects on excessive online behaviors.

	Excessive Gambling						Excessive Gaming						Excessive Internet Use					
	B	SE (B)	95 %	CI	Z	*p*	B	SE (B)	95 %	CI	Z	*p*	B	SE (B)	95 %	CI	Z	*p*
**Within-person effects**																		
Escapism	0.18	0.06	0.06	0.29	2.93	0.003	0.50	0.05	0.41	0.60	10.08	<0.001	0.77	0.14	0.49	1.05	5.41	<0.001
Excessive gambling	-	-	-	-	-	-	0.21	0.04	0.13	0.29	4.93	<0.001	0.53	0.12	0.28	0.77	4.22	<0.001
Excessive gaming	0.23	0.05	0.13	0.33	4.43	<0.001	-	-	-	-	-	-	0.53	0.14	0.25	0.82	3.71	<0.001
Excessive internet use	0.04	0.01	0.02	0.06	4.11	<0.001	0.04	0.01	0.02	0.06	4.03	<0.001	-	-	-	-	-	-
Excessive drinking	0.09	0.04	0.00	0.17	2.08	0.038	−0.03	0.03	−0.09	0.04	−0.79	0.427	0.47	0.11	0.25	0.68	4.29	<0.001
Distress	0.00	0.01	−0.03	0.02	−0.16	0.876	0.02	0.01	−0.01	0.04	1.40	0.162	0.13	0.04	0.05	0.22	3.02	0.003
**Between-person effects**																		
Escapism	0.20	0.19	−0.17	0.56	1.07	0.285	0.91	0.05	0.81	1.02	17.22	<0.001	0.61	0.29	0.04	1.19	2.09	0.036
Excessive gambling	-	-	-	-	-	-	0.14	0.04	0.07	0.22	3.76	<0.001	0.10	0.12	−0.13	0.33	0.87	0.385
Excessive gaming	0.59	0.14	0.32	0.86	4.26	<0.001	-	-	-	-	-	-	1.30	0.19	0.92	1.67	6.73	<0.001
Excessive internet use	0.01	0.02	−0.02	0.05	0.83	0.409	0.04	0.01	0.03	0.06	5.81	<0.001	-	-	-	-	-	-
Excessive drinking	0.13	0.04	0.06	0.21	3.47	0.001	0.00	0.01	−0.03	0.03	0.20	0.840	−0.07	0.08	−0.23	0.10	−0.81	0.419
Distress	0.03	0.02	−0.01	0.07	1.68	0.094	−0.04	0.01	−0.06	−0.02	−4.23	<0.001	0.47	0.07	0.34	0.61	6.94	<0.001
**Controls**																		
Male	0.03	0.15	−0.27	0.33	0.19	0.846	0.28	0.08	0.13	0.43	3.71	<0.001	−1.90	0.42	−2.73	−1.08	−4.52	<0.001
Age	0.02	0.01	0.00	0.03	2.50	0.013	0.00	0.00	−0.01	0.00	−1.41	0.160	−0.14	0.02	−0.18	−0.11	−7.93	<0.001
Master’s degree or higher	−0.30	0.14	−0.57	−0.02	−2.11	0.035	−0.08	0.08	−0.23	0.07	−1.01	0.310	0.59	0.52	−0.43	1.60	1.13	0.258
Working	0.30	0.16	−0.01	0.60	1.89	0.059	−0.02	0.08	−0.18	0.15	−0.21	0.835	−0.69	0.47	−1.61	0.24	−1.45	0.146
High income	−0.06	0.21	−0.31	0.76	−0.47	0.342	−0.04	0.09	−0.23	0.14	−0.45	0.652	−0.19	0.49	−1.15	0.77	−0.40	0.691
In official relationship	−0.12	0.16	−0.76	0.45	−0.42	0.187	−0.05	0.08	−0.20	0.11	−0.59	0.554	−0.63	0.44	−1.50	0.24	−1.41	0.158
Children	0.17	0.17	−0.15	0.50	1.03	0.304	0.10	0.09	−0.07	0.27	1.17	0.243	0.97	0.45	0.09	1.84	2.16	0.031

B = regression coefficients, *p* = *p*-value for statistical significance, Z = z-value for statistical effect.

## Data Availability

The data will be available after completion of the project at Finnish Social Science Data Archive. The data are available from the corresponding author (A.O.) upon reasonable request.
